# Becoming oneself in the other's mirroring

**DOI:** 10.3389/fpsyg.2023.1184770

**Published:** 2023-05-23

**Authors:** Qingming Liu, Huimin Cui, Da Dong

**Affiliations:** ^1^Center for Brain, Mind and Education, Shaoxing University, Shaoxing, China; ^2^Department of Psychology, Shaoxing University, Shaoxing, China

**Keywords:** social interactions, cognitive science, neuroplasticity, cognitive maturity, feelings

To work toward an understanding of how our encounters with others carry us into actions, and continue to shape our actions, and therefore our basic cognitive capacities, we need an account of social interaction (Gallagher, 2020, p. 1).

The human brain is a complex system. Solving the mystery of the brain will require decades or even centuries of joint efforts from many sub-disciplines. Mostly, however, there is a gap between fields and disciplines. In recent years, an increasing number of scientists have begun to study the relationship between innate and acquired knowledge. Nevertheless, the results of these studies have rarely been applied by experts in neighboring disciplines or other fields. Much remains to be done to bridge the interdisciplinary gap and promote collaboration. Neurologists, including neuroscientists, neuropsychologists, and neurophysiologists, do not interact much with educators in neuroscience and pedagogy, and there is a lack of communication between these two fields. Educationalists believe that neuroscience is a laboratory science with little contribute to real-life educational work. Other scholars say that they hope more people will understand how the social environment affects cognitive development. This will make us realize the importance of nurturing relationships, especially for children and adolescents. It is therefore necessary to examine brain development and cognition from a social pedagogy perspective. Isabella (2022) has engaged in interdisciplinary exchanges and interactions with experts in various fields, such as social workers, developmental psychologists, child psychoanalysts, and has raised some of the most important questions about the impact of the social environment on human cognition. In this monograph, *The Making and Breaking of Minds: How Social Interactions Shape the Human Mind*, published in 2022, Sarto-Jackson discusses these issues and explores how social interactions shape the human mind. Social interactions are a two-winged notion. More specially, one aspect is related to intuition in relational behavior, while the other is based on inter-subject verbal exchange that typically is intended.

In addition to the introduction, this book consists of nine chapters. In the first chapter, the author points out that innate genes interact with the acquired environment. In subsequent chapters (Chapters 2–5), the author describes how the environment affects an individual's biological endowment and the cognitive changes that result from socio-environmental experiences. In addition, the book discusses the effects of negative environmental conditions, such as stress and traumatic experiences (Chapter 6), social neglect (Chapter 7), and abuse (Chapter 8), on brain development and cognitive maturity in children and adolescents. The adversities that an individual encounters in their environment have a serious impact on learning, memory, and emotional development. These cognitive developmental biases, in turn, create lifelong influences in terms of emotional attachment, relationships, and social behavior. The author concludes the book by calling for the development of resilience in adolescents, and for adolescents to be put into practice to help them withstand adversity and recover from setbacks.

In the first chapter, the author points out that the innate conditions of an individual interact with the environment. The congenital and acquired struggle for personal development originated in the seventeenth century, and many scientists have strived to solve the problem of what is innate and what is based on learning and experience. Through protracted and unremitting exploration, it has become clear that many individual characteristics are neither predetermined by strict genetic programs nor simply the outcomes of particular environments. On the one hand, the genes of the organism itself affect its own development. On the other hand, individuals constantly adapt to their environment and specific living conditions but also change their environment. In the face of environmental pressures, individuals change their impact by actively changing their environment. Individuals and the environment are linked by an interactive process, constantly influencing and complementing each other. In biology, Odling-Smee et al. (2003) call this causal reciprocity niche construction. This has essential implications for the evolution of human characteristics, especially psychological and spiritual abilities. Human beings change not only the ecological environment, but also the social environment. The author mentions that individuals develop positive traits, but negative outcomes such as anxiety, depression, and narcissistic personality disorder may also follow.

In Chapters 2 and 3, the author discusses neuroplasticity, one of the results of the collision between the environment and talent. Neuroplasticity is a basic property of the brain that operates physiologically at the molecular and cellular levels, as well as under pathological conditions. It refers to adaptive changes in brain circuits caused by neuroplasticity events, resulting in changes in brain function. These changes will be manifested and assessed at the behavioral level. Neuroplasticity begins in infancy, and the fetal and neonatal are ready for subsequent adaptation processes. Neuroplasticity is particularly pronounced in childhood and adolescence. During the maturation of the brain, neuroplasticity gradually decreases. This process is essential for brain development in children and adolescents, and for recovery from neurotrauma. Adolescence is a different stage of brain development, and some scholars describe puberty as a “second individuation process” and a “second chance” (Eissler, 1958, p. 223–254). At this stage, individuals are most vulnerable to constant change due to high plasticity. Unfortunately, this extraordinary brain plasticity is not always positive, as it sometimes makes children and adolescents particularly vulnerable to negative environmental and social factors; moreover, some physical or psychological traumas can greatly interfere with their development. The behavioral patterns acquired by individuals in their previous specific social environments were mapped to their current environment. Individuals adapt to a negative environment in terms of behavior and neuroplasticity, and they also seek similar social environments later in life. This will make it difficult for them to shake off the shadow of past trauma. In general, after puberty, neuroplasticity affects individual development with less change with age. However, the post-traumatic distress syndrome with its acute and chronic manifestations requires our attention. For example, also occur in adulthood upon severely traumatic experiences and are suited to affect the victims' behavior profoundly.

In Chapter 4, the author focuses on gene expression, another direct consequence of the environment impinging on the biological endowment. Neuroplasticity is a form of adaptive plasticity. All organisms face environmental challenges during growth and development. Besides, organisms have adaptive plasticity to overcome these challenges. By undergoing changes, they become better adapted to their living environments. Adaptive plasticity is usually achieved through gene regulatory mechanisms, that is, genes expressed at given times and under specific environmental conditions. Despite having the same combination of genes, the results of gene expression may vary due to the direct effects of the environment; furthermore, individuals may exhibit significant differences in behavior, appearance, or personality traits. While some individuals may have allele variants in their DNA, these genetic variants may increase the likelihood of certain medical disorders, psychopathology, or adverse behavioral traits. However, the same alleles are expressed differently in diverse environments, and what is destructive in one environment may be a key part of success in a healthy and beneficial environment.

Chapter 5 focuses on the memory formation process. Everything we experience, and when and where it happens, is processed in different sensory areas of the cerebral cortex, and eventually formed into memories. The memory process is accomplished through interconnected information transfer between different parts of the brain, which is closely linked to emotional experiences. Thus, the formation of memories involves not only knowledge stored in the hippocampus and other regions of the neocortex but also relies heavily on the emotional processing that occurs in the amygdala. At the psychological level, emotions regulate neuroplasticity, which drives the formation of memories and ensures that certain events or facts are better remembered. As mentioned earlier, neuroplasticity is particularly pronounced during childhood and adolescence, when the human brain is undergoing a great deal of shaping and reorganization. However, the brain is also susceptible to adverse environmental influences during development, impairing an individual's cognitive development, emotional processing, and executive brain function.

In Chapter 6, the author discusses the effects of emotions, stress, and traumatic experiences on cognition. In addition to their key role in memory, emotions determine how a person perceives the world, makes important decisions, and constructs identity. A person's entire identity is constantly renewed as new knowledge and memories are integrated into an existing, emotionally stimulated self-image. In addition, the neural processes of believing that are fundamental for integrating environmental influences and innate emotional conditions underlying decision making and social interactions (Seitz et al., 2022). Memories are usually stored associatively and are integrated into neural networks based on previous experience. However, traumatic events are often highly unexpected and abrupt experiences, and it is difficult for victims to integrate this unique but negative experience into the existing framework of experience. It is also difficult to integrate the traumatic experience into a person's coherent and enduring sense of self and world. Consequently, they may not be able to relate their current behavior to their future selves and recognize their own behavior and the consequences that come with it (Quartz and Sejnowski, 2002). When survivors who have experienced traumatic events during the developmental period later experience similar situations again, they fall back into a reactive state of self-preservation and survival behavior, producing a strong overreaction. The role of the prefrontal cortex as the “executive” center of brain. The prefrontal cortex synthesizes information from various other brain regions. Traumatic experiences, abuse, and neglect strongly interfere with the adaptive shaping of frontal cortex function during childhood brain development, resulting in a reorganization of developing neural networks in the prefrontal cortex, which impairs decision-making and self-control. Damage to the connection from the amygdala and the prefrontal cortex affects emotional processing in individuals, emotionally frustrated people may respond violently to stress responses and become abusive. They recreate situations that are emotionally similar to what they experienced when they were previously traumatized. In this environment, abuse continues and is passed on socially to the next generation.

Chapter 7 focuses on the impact of parenting styles and attachment on children's emotional and cognitive development. Children's stress systems are not yet fully mature in infancy and, therefore, cannot effectively regulate stress responses. Children are highly dependent on their social environment to calm their excessive physical stress response through the comfort of their parents or other caregivers. The physical and psychological stress response to social isolation resulting from the lack of parent-child interaction is particularly acute in children and adolescents. They are predominantly vulnerable due to immature neurons and hormonal stress response systems, which cause long-term damage to brain structure and function. Therefore, mother–child attachment is essential for a child's healthy emotional and cognitive development. Developmental psychologists divide attachment styles according to the impulses that develop in infancy in response to maternal attitudes. Attachment styles are formed in infancy and further strengthened or modified in childhood, adolescence, and adulthood. Different styles describe how people connect with and interact with others. Secure attachment develops when children feel that their parents are responsive to their needs and are willing to communicate with them. Conversely, children with a history of neglect or high-risk situations develop other undesirable attachment styles. Poor attachment styles can cause difficulty in maintaining intimacy in children as adults. Behaviorists believe that there is a way to raise children to cope with the realities of modern life. The attachment theory emphasizes that the mother–child union is a necessary evolutionary survival strategy for the healthy development of children. Mother–child attachment regulates behavior and, provides environmental adaptation. However, parents are likely to convey information about the quality of their children's environment through their parenting style. Therefore, there is no single ideal parenting style because different environmental needs require offspring to have different characteristics that are enhanced by parental care.

In Chapter 8, the author focuses on the pernicious effects of violence on individual development. Some adults who have experienced abuse, emotional neglect, and suffering their childhood will be haunted by their experiences later in life. The available developmental trajectories are severely limited, often leading to personality problems and manifestations of psychopathological symptoms. Notably, parents who are also victims of domestic violence are at a higher risk of committing violence against their children, severely interfering with their children's development of safe attachment styles. When they become parents, they carry their adverse childhood experiences into their adult lives and new environments. Childhood experiences shape the inner psychological structure while forming a template for social relationships with others. Humans live in social environments, and the economic conditions associated with them determine material resources, broader social interactions, and parental investment. Often, access to social support depends largely on the social environment in which children grow up. Improving socio-economic conditions is therefore one of the most important levers for children's positive development.

The last paragraph emphasizes the mental resilience. After experiencing unspeakable fear and abuse, there are some people who are able to overcome their trauma and lead happy lives. American developmental psychologist Werner and Smith (1998) calls this characteristic of children who thrive despite a high-risk developmental history “resilience.” This has led to the emergence of studies on mental resilience. For children and adolescents experiencing severe adversity, providing a safe environment, and working to meet their personal growth needs can help build resilience and overcome adversity. Martin Brokenleg and Larry Brendtro developed the “Circle of Courage,” a holistic approach to helping at-risk children and adolescents. The “Circle of Courage” depicts the four developmental needs of all children: belonging, mastery (self-esteem), independence (self-actualization), and generosity (self-transcendence). It builds resilience in young people by linking wellbeing, behavior, and learning, shifting the focus from weaknesses to strengths (Brendtro, 2019, p. 5–24). In addition, because children learn by imitating adults, opportunities must be provided to observe and learn how to continuously cooperate, help, and care for others with adults.

In summary, this book aims to provide up-to-date interdisciplinary knowledge between neuroscience, biopsychology, and social pedagogy, which experts from neighboring disciplines can use for psychoeducation. However, the book has certain limitations. The author focuses on “how social interactions shape the human mind,” without scientific exploration at the molecular and neurobiological level, as well as information processing, neural networks, mental states and cognition. Certainly, as a tool in psychoeducation, the book highlights multiple aspects of the interaction between innate and acquired factors, while emphasizing the importance of psychosocial factors for development. As discussed in the book, developmental trajectories are shaped by a variety of biological, social, environmental, and economic factors. At this critical stage of development in children and adolescents, individuals exhibit strong adaptive plasticity and are susceptible to positive or negative influences. Traumatic events experienced in childhood can affect the lifelong development of an individual and even have an intergenerational impact. Creating an environment that build resilience in children and adolescents can help them to overcome hardships and create a happy life.

## Author contributions

QL and HC wrote the manuscript. DD is in charge of the idea. All authors listed have made a substantial, direct, and intellectual contribution to the work and approved it for publication.
